# Sweet Saboteur: Insulinoma Presenting As Recurrent Hypoglycemic Seizures

**DOI:** 10.7759/cureus.63205

**Published:** 2024-06-26

**Authors:** Jayaram Saibaba, S Siyamala, Gopinath Karuppiah, Soundarya Ravi, Kolar V Vinod

**Affiliations:** 1 General Medicine, Jawaharlal Institute of Postgraduate Medical Education & Research, Pondicherry, IND; 2 Pharmacology and Therapeutics, Jawaharlal Institute of Postgraduate Medical Education & Research, Pondicherry, IND; 3 Medicine, Jawaharlal Institute of Postgraduate Medical Education & Research, Puducherry, IND; 4 Pathology, Jawaharlal Institute of Postgraduate Medical Education & Research, Pondicherry, IND; 5 General Medicine, Jawaharlal Institute of Postgraduate Medical Education & Research, Puducherry, IND

**Keywords:** multiple endocrine neoplasia type 1 (men-1), octreotide, pancreatic neuroendocrine tumor, whipple's triad, endogenous hyperinsulinemia, neuroglycopenic symptoms, hypoglycemic seizures, insulinoma

## Abstract

Insulinoma, a rare neuroendocrine tumor of the pancreas, often presents diagnostic challenges due to its diverse clinical manifestations. We present the case of a 25-year-old female with recurrent hypoglycemic seizures and neuroglycopenic symptoms, ultimately diagnosed with insulinoma. Despite an initial asymptomatic period, the patient experienced progressively worsening symptoms over three years, culminating in eight episodes of generalized tonic-clonic seizures per week. Biochemical investigations during hypoglycemic episodes revealed elevated C-peptide and insulin levels, consistent with endogenous hyperinsulinemia. Imaging studies, including contrast-enhanced computed tomography (CECT) and Ga-DOTATATE scan, confirmed a hyper-enhancing lesion in the distal body of the pancreas, indicative of insulinoma. Histopathological examination (HPE) further corroborated the diagnosis. Prompt recognition and surgical excision led to the complete resolution of symptoms and improved long-term prognosis. This case underscores the importance of considering insulinoma in young individuals presenting with recurrent hypoglycemic episodes and highlights the significance of early diagnosis and intervention in preventing morbidity and mortality associated with this condition.

## Introduction

Insulinoma is the most common functional neuroendocrine tumor of the pancreas, and it secretes insulin, leading to hypoglycemia. The prevalence is 1 to 4 per million in the general population [1-5]. Most insulinomas are located in or attached to the pancreas, and the most common extrapancreatic location is in the duodenal wall [5]. They can occur at any age and equally in both males and females. More than 90% of them are solitary, benign, occur at intrapancreatic sites, and size <2 cm in diameter [6-9]. 

In this case report, we highlight a young female who presented with recurrent hypoglycemic seizures and neuroglycopenic symptoms and was found to have endogenous hyperinsulinemia, fitting Whipple's triad, and imaging and histopathological examination (HPE) suggestive of insulinoma. Medical treatment was used to abort the hypoglycemic episode, and after the localization of the lesion, the definitive treatment was surgical excision of the tumor.

## Case presentation

Twenty-five-year-old female with no known comorbidities, working in a bottle company, residing at Vadalur, symptomatic for three years, presented with giddiness, palpitation, sweating, and recurrent seizures (eight episodes). 

The patient was asymptomatic three years before, then she had insidious onset, gradually progressive, recurrent episodes of giddiness, increased over one month, associated with tremors and sweating, especially towards early morning. Following this, she had eight episodes of generalized tonic-clonic seizures presenting with involuntary tonic-clonic movements of all four limbs and a loss of consciousness, lasting for two to three minutes. No tongue bite, urinary incontinence, or postictal confusion. During the time of seizures, sugars were documented outside (40 mg/dl). On giving oral glucose, patient symptoms and sensorium improved. History of weight gain of 3 kg in the past six months to the present. No history of post-prandial hypoglycemia, vomiting, abdominal pain, abdominal distention, burning micturition, jaundice, altered bowel habits, fever, headache, vomiting, blurred vision, bleeding manifestations, joint pain, or rash. No history of any other drug intake except for iron supplements. No history of previous surgery/gastric bypass surgeries. Outside evaluation for seizures (non-contrast CT brain and electroencephalogram (EEG)) was normal. No family history of similar complaints; however, her mother is diabetic, diagnosed at 50 years of age. She has two elder sisters and one younger brother. She has two children - one boy (five years) and one girl (three years). The last childbirth was in 2008. There were no other complaints.

**Figure 1:**
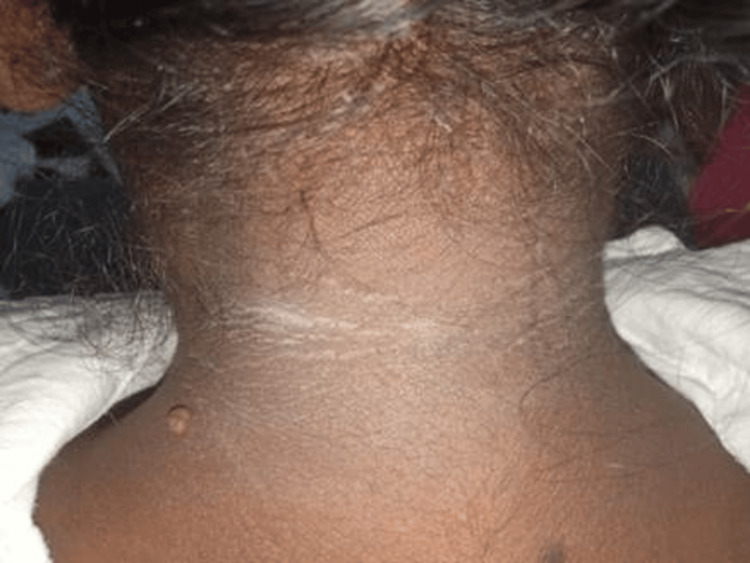
Acanthosis nigricans at the posterior aspect of the neck

**Figure 2:**
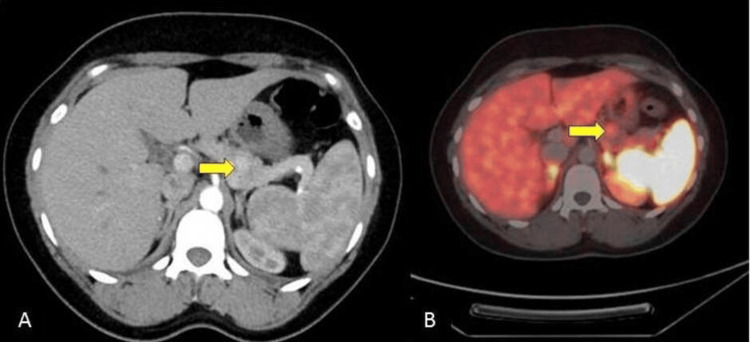
A: Contrast-enhanced CT of the abdomen showing 1.6x1.5x1.2 cm hyper-enhancing lesion in arterial phase in the distal body of pancreas (marked by arrow); B: Ga-DOTATATE scan showing an increased uptake by lesions in the distal body of pancreas suggestive of insulinoma (marked by arrow) At a standardized uptake value of 2.19

**Figure 3:**
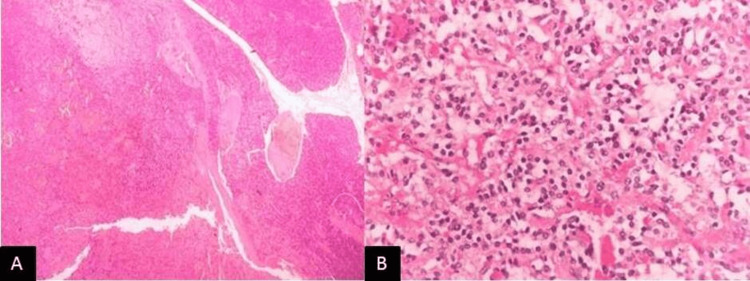
A: lower power 40x H&E and B: higher power 400x H&E magnification of a grade 2 well-differentiated neuroendocrine tumor arising from the pancreas, composed of monomorphous tumor cells arranged in nests and trabecular pattern, exhibiting moderate amounts of eosinophilic cytoplasm, round nuclei with stippled chromatin

**Figure 4:**
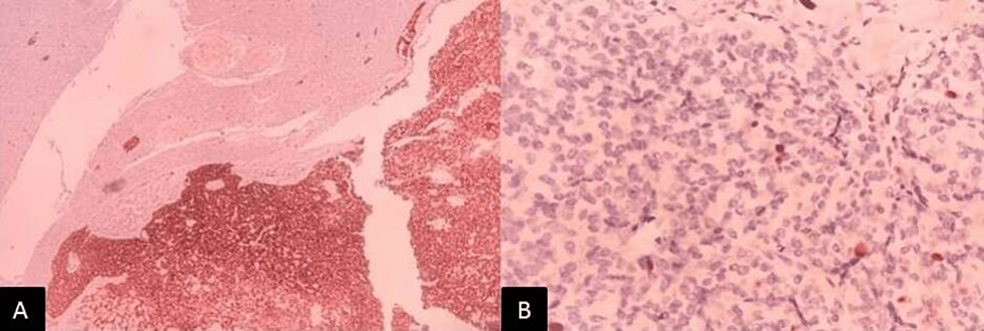
On immunohistochemistry, the tumor cells (insulinoma rests) are highlighted by synaptophysin (A: 100x; 3,3' diaminobenzidine) with Ki67 labeling index of 5% (B: 400x; 3,3' diaminobenzidine)

On examination, the patient was conscious, oriented, and afebrile. The patient's vital signs were within normal limits: the pulse rate was 84/min, blood pressure was 110/70 mm Hg, respiratory rate was 18/min, and saturation (SpO2) was 98%. The patient was 160 cm tall, weighed 60 kg, with a body mass index (BMI) of 23.43 kg/m², and exhibited acanthosis nigricans (Figure 1). Pallor was present, while there was no icterus, cyanosis, clubbing, lymphadenopathy, or pedal edema. A systemic examination was unremarkable. All blood investigations and hormone panels are listed in Table 1. It shows elevated serum fasting insulin levels and C-peptide levels towards the upper limit of normal. Endogenous hyperinsulinemia causes cholinergic/ neuroglycopenic symptoms, and recurrent episodes of hypoglycemic seizures fit Whipple's triad, suggestive of insulinoma. Contrast-enhanced computed tomography (CECT) of the thorax, abdomen, and pelvis showed hyper-enhancing lesions in the arterial phase in the distal body of the pancreas (Figure 2A), confirmed by Ga-DOTATATE scan with increased standardized uptake value suggestive of insulinoma (Figure 2B). Workup for multiple endocrine neoplasia-1 (MEN-1) in our patient was negative. The patient and her attendees were educated regarding the symptoms of hypoglycemia and how to diagnose and treat it. The patient was counseled regarding self-monitoring of blood glucose and glucose charting. The patient was initially treated with somatostatin analog octreotide 200 mcg/day into two divided doses. She was also treated with oral diazoxide 3 mg/kg/day divided into three equal doses every eight hours. However, the definitive treatment is surgery. After the localization of the tumor to the distal body of the pancreas, laparoscopic distal pancreatic splenectomy was done under general anesthesia. Intraoperative findings revealed no free fluid in the abdominal cavity, and the liver surface was normal. The pancreas was soft in consistency, with no surrounding inflammation. Intraoperative ultrasound (IOUS) showed a well-defined hypoechoic lesion in the body of the pancreas. The rest of the pancreas was normal. The main pancreatic duct was not dilated. Post-procedure, the patient's vitals were stable, and she improved completely post-surgery with no further hypoglycemic episodes or neuroglycopenic episodes. Histopathological examination of the specimen showed lower power (40x H&E; Figure 3A) and higher power (400x H&E; Figure 3B) magnification of grade 2 well-differentiated neuroendocrine tumor arising from the pancreas, composed of monomorphous tumor cells arranged in nests and trabecular pattern, exhibiting moderate amounts of eosinophilic cytoplasm, round nuclei with stippled chromatin. On immunohistochemistry, the tumor cells are highlighted by synaptophysin (100x; 3,3' diaminobenzidine; Figure 4A) with a Ki67 labeling index of 5% (400x; 3,3' diaminobenzidine; Figure 4B). After surgery, the patient improved significantly; no hypoglycemic episodes recurred, and the patient was doing well at six months follow-up. 

**Table 1:** Blood investigations and entire hormonal panel of the patient Hb - hemoglobin; WBC - white blood cells; MCV - mean corpuscular volume; PS - peripheral smear; AST - aspartate aminotransferase; ALT - alanine aminotransferase; ALP - alkaline phosphatase; GGT - gamma-glutamyl transferase; TSH - thyroid stimulating hormone; PTH - parathyroid hormone

Parameters	Values	Reference values
Complete blood count		
Hemoglobin	8.7 g/dl	11.7-15.5 g/dL
White blood counts	8.30 x 10^3^/uL	4.0-11.0 x 10^3^/uL
Neutrophil	76%	40-75%
Lymphocyte	20.1%	20-45%
Monocytes	3.4%	2-10%
Platelets	278 x 10^3^/uL	150-450 x 10^3^/uL
Mean corpuscular volume	66.9 fL	80-100 fL
Peripheral smear	Microcytic hypochromic blood picture	
Ferritin	22.3 ng/ml	13-150 ng/ml
Iron	15 mcg/dL	60-170 mcg/dL
Renal function test		
Urea	7 mg/dl	17-43 mg/dl
Creatinine	0.63 mg/dl	0.5-0.95 mg/dl
Sodium	138 mEq/L	136-146 mEq/L
Potassium	3.8 mEq/L	3.5-5.1 mEq/L
Calcium	8.7 mg/dl	8.8-10.6 mg/dl
Magnesium	2.8 mg/dl	1.9-2.5 mg/dl
Liver function test		
Total bilirubin	0.30 mg/dL	0.3-1.2 mg/dL
Direct bilirubin	0.69 mg/dL	0.03-0.18 mg/dL
Serum total protein	6.6 mg/dL	6.6-8.3 g/dL
Serum albumin	3.5 mg/dL	3.5-5.5 g/dL
AST	24 IU/L	0-35 IU/L
ALT	12 IU/L	0-35 IU/L
ALP	63 IU/L	7-56 IU/L
GGT	15 IU/L	5-40 IU/L
Amylase	30 IU/L	40-140 IU/L
Lipase	16 IU/L	0-160 IU/L
Triglycerides	130 mg/dL	<150 mg/dL
Free T3	2.78 pg/ml	2.3-4.2 pg/ml
Total T4	7.7 ug/dL	6.5-12 ug/dL
TSH	3.14 uIU/ml	0.35-5.50 uIU/ml
Cortisol	8.31 ug/dL	4.3-22.4 ug/dL
Prolactin	6.34 ng/ml	2.8-29.2 ng/ml
PTH	39 pg/ml	18.3-80.1 pg/ml
C-peptide levels during the hypoglycemic episode (at critical blood glucose level of 40 mg/dl)	5.17 ng/ml	0.78-5.19 ng/ml
Serum insulin levels during the hypoglycemic episode (at critical blood glucose level of 40 mg/dl)	49.7 uIU/ml	2-25 uIU/ml

## Discussion

The beta islet cell insulin-secreting tumours represent 10-30% of all pancreatic tumours with MEN-1. It fits Whipple's triad, and biochemical investigations taken during the hypoglycemic episode reveal elevated C-peptide levels and insulin levels. It can occur with gastrinoma in 10% of patients of MEN-1. The first episode of hypoglycemia must be recognized and treated early to prevent hypoglycaemic unawareness and autonomic failure in subsequent attacks. After ruling out common causes of hypoglycemia - drugs, critical illness, hormonal deficiency, and dumping syndrome - the patient must be evaluated for endogenous hyperinsulinism/islet cell tumors [2]. Close monitoring and treating hypoglycemic episodes [10], medical management to abort the episode, and definitive management is surgical excision. Post-surgical excision, there is a complete resolution of symptoms, no further episodes of neuroglycopenic episodes, and improved long-term survival [11]. Insulinoma can present with recurrent hypoglycemic seizures, and it is very crucial to check for serum insulin and C-peptide levels during the hypoglycemic episode (critical blood glucose level); it is a totally reversible or correctable condition after localizing and resection of the lesion. For preventing acute episodes, octreotide and diazoxide are used. Verapamil has been shown to inhibit insulin release and reduce the incidence of hypoglycemic episodes, and it can be tried in refractory cases. All these patient needs to be worked up for MEN-1 syndrome and need further serial follow-up in the future.

## Conclusions

This case highlights the need to have a high index of suspicion of insulinoma with respect to recurrent hypoglycemic episodes in young females having endogenous hyperinsulinemia and fitting Whipple's triad. Insulinoma is a treatable condition. Early diagnosis of the hypoglycemic episode is very crucial as recurrent episodes can precipitate hypoglycemia-associated autonomic failure (HAAF), leading to the presentation of neuroglycopenic symptoms with only a very low threshold of glucose. Not all seizures are purely neurological; it's important to consider metabolic factors as well. Any patient with recurrent hypoglycemic seizures or neuroglycopenic features is to be evaluated for insulinoma, and future follow-up for MEN-1 syndrome evaluation is necessary. Biochemical investigations (C-peptide, serum insulin, and corresponding critical blood glucose level) levels at the time of hypoglycemic episodes help in diagnosis. Medical management with octreotide and diazoxide is for aborting acute episodes, and surgical excision of the lesion is definitive management in these cases. Early recognition, diagnosis, and timely treatment can save the life of patients. 
